# Risk of preterm birth for placenta previa or low-lying placenta and possible preventive interventions: A systematic review and meta-analysis

**DOI:** 10.3389/fendo.2022.921220

**Published:** 2022-09-02

**Authors:** Charlotte H. J. R. Jansen, Charlotte E. van Dijk, C. Emily Kleinrouweler, Jacob J. Holzscherer, Anouk C. Smits, Jacqueline C. E. J. M. Limpens, Brenda M. Kazemier, Elisabeth van Leeuwen, Eva Pajkrt

**Affiliations:** ^1^ Department of Obstetrics and Gynecology, Amsterdam University Medical Centers (UMC), University of Amsterdam, Amsterdam, Netherlands; ^2^ Amsterdam Reproduction and Development Research Institute, Amsterdam, Netherlands; ^3^ Department of Research Support–Medical Library, Amsterdam UMC, University of Amsterdam, Amsterdam, Netherlands

**Keywords:** placenta previa, low-lying placenta, preterm birth, cerclage, pessary, progesterone, preventive interventions

## Abstract

**Objective:**

To investigate the risk of preterm birth in women with a placenta previa or a low-lying placenta for different cut-offs of gestational age and to evaluate preventive interventions.

**Search and methods:**

MEDLINE, EMBASE, CENTRAL, Web of Science, WHO-ICTRP and clinicaltrials.gov were searched until December 2021. Randomized controlled trials, cohort studies and case-control studies assessing preterm birth in women with placenta previa or low-lying placenta with a placental edge within 2 cm of the internal os in the second or third trimester were eligible for inclusion. Pooled proportions and odds ratios for the risk of preterm birth before 37, 34, 32 and 28 weeks of gestation were calculated. Additionally, the results of the evaluation of preventive interventions for preterm birth in these women are described.

**Results:**

In total, 34 studies were included, 24 reporting on preterm birth and 9 on preventive interventions. The pooled proportions were 46% (95% CI [39 – 53%]), 17% (95% CI [11 – 25%]), 10% (95% CI [7 – 13%]) and 2% (95% CI [1 – 3%]), regarding preterm birth <37, <34, <32 and <28 weeks in women with placenta previa. For low-lying placentas the risk of preterm birth was 30% (95% CI [19 – 43%]) and 1% (95% CI [0 – 6%]) before 37 and 34 weeks, respectively. Women with a placenta previa were more likely to have a preterm birth compared to women with a low-lying placenta or women without a placenta previa for all gestational ages. The studies about preventive interventions all showed potential prolongation of pregnancy with the use of intramuscular progesterone, intramuscular progesterone + cerclage or pessary.

**Conclusions:**

Both women with a placenta previa and a low-lying placenta have an increased risk of preterm birth. This increased risk is consistent across all severities of preterm birth between 28-37 weeks of gestation. Women with placenta previa have a higher risk of preterm birth than women with a low-lying placenta have. Cervical cerclage, pessary and intramuscular progesterone all might have benefit for both women with placenta previa and low-lying placenta, but data in this population are lacking and inconsistent, so that solid conclusions about their effectiveness cannot be drawn.

**Systematic review registration:**

PROSPERO https://www.crd.york.ac.uk/prospero/, identifier CRD42019123675.

## Introduction

Women with a placenta previa, overlying the internal os of the cervix, or a low-lying placenta, within 20 millimeter (mm) of the internal os of the cervix, have an increased risk of maternal and fetal complications during pregenancy (1, 2). The most important fetal complication is preterm delivery, which is the leading cause of neonatal morbidity and mortality (3–7). An estimated 11% of all world’s live births are preterm, whereas in women with placenta previa or a low-lying placenta this risk has been reported to be 2 to 4 times increased (8, 9). It has been speculated, that due to poor blood flow in the lower uterine segment and enlargement of the lower uterine segment in the third trimester, low-lying placentas detach more easily from the underlying decidua basalis. This can trigger a cascade of events ensuing vaginal bleeding, contractions, cervical effacement and dilation subsequently leading to preterm birth (1, 10–14). Thus, preterm births in women with placenta previa or a low-lying placenta are often caused by emergency deliveries for severe blood loss either with or without spontaneous onset of contractions. However, in order to avoid preterm abundant blood loss, they may be predominantly caused by scheduled cesarean sections before 37 weeks of gestation. Consequently, there is an increased risk of placenta previa in a subsequent pregnancy, as both a cesarean section in the obstetric history and placenta previa are both significant risk factors (15–19).Other known risk factors are multiple gestation, previous uterine surgical procedures, pregnancy termination or uterine artery embolization, increasing maternal age and parity, smoking, cocaine abuse and male fetus, of which some are independent (20–22). Especially given the increasing numbers of cesarean sections worldwide, it is important to consider this consequence as well to avoid future problems. Notably, in the case of a low-lying placenta, a trial of labor is often recommended unless major (bleeding) complications are present (2, 14, 23).

The risk of neonatal morbidity and mortality depends mainly on the severity of the prematurity (9, 24). It is important to counsel future parents considering the risks of preterm birth at different gestational ages and to consider preventive interventions. Therefore, we aim to review the current literature on the risk of preterm birth before 37, 34, 32 and 28 weeks of gestation in women with a placenta previa and in women with a low-lying placenta and to compare this between the two groups.

To a greater and lesser extent, cerclage, pessary and progesterone are known interventions that prolong gestation in women at high-risk of preterm delivery (25–27). Yet, the effectiveness of these preventive methods is largely unknown for pregnancies complicated by placenta previa or low-lying placentas (28). Therefore, in addition, we evaluate and describe the reported effect of intervention to preterm birth in this group of women.

## Methods

### Study design and systematic review protocol

This systematic review was reported according to the Preferred Reporting Items for Systematic Reviews and Meta-analyses (PRISMA) statement (**Supplementary Information 1**) (29). The review protocol was registered in the prospective register of systematic reviews (PROSPERO: systematic review record CRD42019123675). This systematic review did not receive any specific grant from funding agencies in the commercial, not-for-profit or public sectors. As this article is a review, there was no direct patient- and public involvement in the study.

### Participants, interventions and comparators

Women with a placenta previa or a low-lying placenta are the subject of this systematic review. When possible, we compared women with placenta previa to women without placenta previa or with women with a low-lying placenta. All possible preventive interventions for preterm birth described for these women were evaluated.

### Search strategy and data sources

An information specialist (JL) performed a systematic search in OVID MEDLINE, OVID EMBASE, Web of Science, the Cochrane Controlled Register of Trials (CENTRAL) and the prospective trial registers clinicaltrials.gov and WHO-ICTRP from inception to December 6th, 2021. The search strategy consisted of controlled terms, including MESH-terms, and text words for placenta previa or low-lying placenta and preterm birth. Randomized controlled trials, cohort studies and case-control studies assessing preterm birth in women with placenta previa or low-lying placenta of the internal os in the second or third trimester were eligible for inclusion. Animal studies, conference abstracts, reviews and case reports were (safely) excluded if appropriate. We applied no date or language restrictions. We cross-checked the reference lists and the citing articles of the identified relevant papers in Web of Science and adapted the search in case of additional relevant studies. The bibliographic records retrieved were imported and de-duplicated in ENDNOTE. To evaluate the interventions a similar search strategy was performed on interventions, e.g. cerclage, pessary and progesterone to prevent preterm birth in women with placenta previa or low-lying placenta.

### Study selection

Articles were selected in a staged process using the electronic screening tool RAYYAN. Two reviewers (CJ, CvD) independently screened titles and abstracts of all retrieved articles and selected potentially eligible studies. Both reviewers then independently examined the full-text papers. Disagreements about inclusions were resolved by consensus or by consulting a third reviewer (CK). Over the years, various definitions have been used for placentas that are implanted in the lower part of the uterus such as; minor and major placenta previa, placenta previa totalis, placenta previa partialis, placenta previa marginalis and low-lying placenta. In this review, we have used the currently recommended definitions for placenta previa, overlying the internal os of the cervix, and low-lying placenta, not overlying but within 20 mm of the internal os (30, 31). Aiming for the least heterogeneity between the included studies, we excluded articles that did not define placenta previa and/or low-lying placenta as such. Articles using the older terms of placenta previa partialis were considered as placenta previa, and placenta previa marginalis were considered as low-lying placenta. Studies that reported only the mean or median gestational age at birth were excluded. Gestational age at diagnosis, sonographer skills, types of equipment and standardized protocols for measuring placenta previa or low- lying placenta were not considered as exclusion criteria.

### Data extraction

A predesigned data extraction form was used by two independent reviewers to retrieve study characteristics from each included study. For studies on risk of preterm birth, extracted preterm birth outcomes were proportions (based on given numbers of women with preterm birth and the total number of women) or odds ratios with 95% confidence intervals for preterm birth below 37, 34, 32 and 28 weeks of gestation, or gestational age at delivery. Data was extracted separately for women with a placenta previa or a low-lying placenta. For intervention studies, we extracted data on gestational age at delivery, prolongation of gestation (i.e. the time from randomization to delivery), or odds ratios with 95% confidence intervals for preterm birth.

### Data synthesis and analysis

Results of all studies reporting a proportion (or percentage, rate, incidence) of preterm birth before 37, 34, 32 and/or 28 weeks of gestation were pooled for women with placenta previa and for women with a low-lying placenta, as appropriate based on available data. Analyses of pooled proportions were performed using inverse of the Freeman-Tukey double arcsine transformation using *metafor* and *meta* packages in Rstudio version 3.6.1.For studies reporting the results for more than one group, we compared women with placenta previa to women without placenta previa or with women with a low-lying placenta. Odds ratios (ORs) with 95% confidence intervals (CI) were calculated for the risk of preterm birth before 37, 34, 32, and 28 weeks of gestation.

To illustrate the effect of the interventions to prevent preterm birth in women with a placenta previa described in our overview, we calculated ORs with 95% CI for the risk of preterm birth or mean differences with 95% CI for gestational age at delivery and prolongation of gestation for women with and without preventive interventions (cerclage, pessary, progesterone). The meta-analyses were conducted using Review Manager version 5.3 from the Cochrane Collaboration. In all meta-analyses, a random-effects model was used.

### Assessment of risk of bias

The quality of the studies was assessed with the Newcastle Ottawa scale. Articles with seven or more stars were found to be of high quality, four to five stars have moderate quality and articles with three or less stars were found to be of low quality. The Cochrane Handbook was used to judge the quality of each randomized controlled trial. To assess publication and small study bias, funnel plots were conducted, when at least 10 studies are available, because with fewer studies the power of the test is too low to distinguish chance from true asymmetry (Cochrane Handbook).

## Results

### Study selection and characteristics

**Figure 1:**
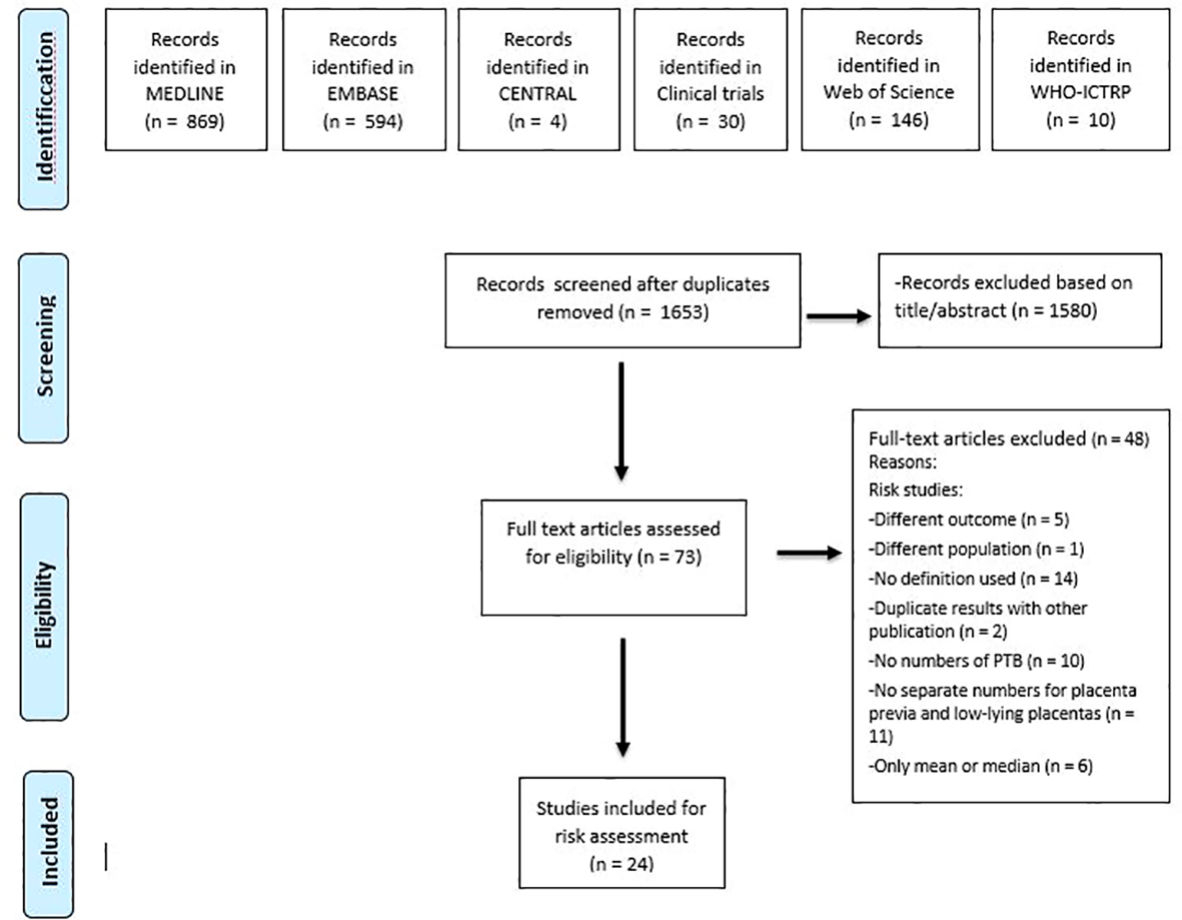
Flow diagram showing selection of studies reporting on risk of preterm birth in women with placenta previa and/ or low-lying placenta and of studies reporting on interventions preventing preterm birth in women with placenta previa.

The search identified 1653 unique records. Of these, 24 were included based on full-text (4, 32–54). **Figure 1** shows the flow-chart. All 24 studies reported on preterm birth in women with a placenta previa and 7 of the articles additionally reported on women with a low-lying placenta (35, 36, 40–42, 45, 49). **Table 1** provides the baseline characteristics for the preterm birth studies.

**Table 1:** Article characteristics for studies on risk of preterm birth.

Author, year, country	Study design	Inclusion period	-Study population-Time of diagnosis	Study groups (N)Cases Controls	Definition cases	Outcome of interest
**Ananth et al, 2003. USA** (32)	Retrosp cohort	1989-1991 1995-1997	-Women with CD -≥ 24 wks, confirmed during CD	PP (61,711) NPP (22,306,524)	PP: placental disc is covering the IO	PTB (n/N)
**Adere et al, 2020. Ethiopia** (33)	Retrosp cohort/unmatched case-control study	Sep 2015 – Jan 2018	-All deliveries/women with PP - the second and third trimesters of pregnancy or intraoperatively	PP (303) NPP (303)	PP: overlying the IO (to any degree)	PTB (n/N)
**Bahar et al, 2009. Saudi Arabia** (34)	Retrosp cohort	Jan 1996 - Dec 2005	-Women with CD -≥ 24 wks, confirmed during CD	PP (173) (major) LLP (133) (minor)	Major: Partially or completely covering the IO Minor; Reaching the IO	PTB (n/N)
**Baumfeld et al, 2017. Israel** (35)	Retrosp cohort	Jan 1998 – Dec 2013	-All deliveries -During delivery	PP (1249) NPP (294,697)	PP: Partially or completely covering the IO	PTB (n/N)
**Bi et al, 2021, China** (36)	Retrosp cohort	Jan 2009 – Jan 2019	-Women with PP ->16 wks, confirmed during delivery	PP (3898) LLP (466)	PP: Completely or partially covering the IO LLP: within 20mm	PTB (n/N)
**Fan et al, 2019. China** (37)	Prosp cohort	Mar 2016 – July 2017	-Women with PP -≥ 28 wks, confirmed during CD	PP (100)	PP: Completely covering the IO	PTD (n/N) GAD (Mean, SD)
**Fishman et al, 2011. USA** (38)	Retrosp cohort	Jan 2002 – Jan 2010	-Women with PP ≥28 wks -confirmed during delivery	PP (113)	PP: Partially or completely covering the IO	PTB (n/N) GAD (Mean, SD)
**Fung et al, 2011. China** (39)	Retrosp cohort	2000 – 2007	-Women with US between 14 – 23 wks -14-23 wks	PP (609) NPP (15627)	PP: Completely covering the IO	PTB (n/N)
**Grgic et al, 2004. Bosnia and Herzegovina** (40)	Retrosp case control	2001 – 2002	-Cases: Women with PP -Not mentioned	PP (12) LLP (4) NPP (16)	PP: partially or completely covering the IO LLP: Inserted close by the IO	PTB (n/N)
**Jauniaux et al, 2019. UK** (41)	Retrosp cohort	6 year period	-Women with PP -Between 20 – 36 wks	PP (146) LLP (64)	PP: <0.5mm of internal os LLP: 0.5mm-20mm of IO	PTB (n/N) GAD (Mean, SD)
**Kollmann et al, 2016. Austria** (42)	Retrosp cohort	1993-2003 2003-2012	-Women with PP -Not mentioned	PP (91) LLP (117)	PP: Completely covering the IO LLP: within the lower uterine segment	PTB (n/N)
**Norgaard et al, 2012. Denmark** (43)	Case-control	2001 – 2006	-Women with CD -Third trimester or during CD	PP (1147) PP + LLP (1721) NPP (8603)	PP + LLP: ICD 10 O44.0-3 and ICD 10 O44.9	PTB (n/N) GAD (Mean, SD)
**Olive et al, 2005. Australia** (44)	Retrosp cohort	July 1998 – Dec 2002	-Women who gave birth -CD >26 wks	PP (1612) NPP (374178)	PP: ICD 10 O44.1	PTB (n/N)
**Ozer et al, 2017. Turkey** (45)	Retrosp cohort	Jan 2004 – Dec 2015	-Women with PP -Just before CD	PP (97) LLP (84)	PP: Completely or partially covering the IO LLP: within 20mm	PTB (n/N)
**Roh et al, 2018. South Corea** (46)	Retrosp cohort	Mar 2010 – Oct 2017	-Women with PP -> 20 wks and CD > 23 wks	PP (140)	PP: Completely or partially covering the IO	PTB (n/N)
**Rosenberg et al, 2011. Israel** (4)	Retrosp cohort	1988-2009	-Women with and without PP -2^nd^ and 3^rd^ trimester	PP (771) NPP (184,705)	PP: Completely or partially covering the IO	PTB (n/N)
**Roustaei et al, 2018. Finland** (47)	Retrosp cohort	2004 – 2008	-Pregnant women -2^nd^ and 3^rd^ trimester	PP (714) NPP (282,609)	PP: Covering the IO	PTB (n/N) GAD (Mean, SD)
**Ruiter et al, 2016. Netherlands** (48)	Retrosp cohort	Jan 2001 – Dec 2011	-Women with PP and scheduled CD ->24 wks	PP (214)	PP: Within 20 mm	PTB (n/N)
**Sekiguchi et al, 2013. Japan** (49)	Retrosp cohort	Jan 2004 – Mar 2012	-Women with PP -3^rd^ trimester	PP (71) LLP (91)	PP: Completely covering IO LLP: lying close by the IO	PTB (n/N)
**Sheiner et al, 2001. Israel** (50)	Retrosp cohort	1990 – 1998	-Women delivering -Confirmed during delivery	PP (298) NPP (78,226)	PP: Completely covering the IO in the third trimester	PTB (n/N)
**Weiner et al, 2016. Israel** (51)	Retrosp cohort	Jan 2009 – Dec 2015	-Women with PP between 24 – 42 wks -Confirmed at CD	PP (137)	PP: Partially or completely covering the IO	PTB (n/N)
**Yeniel et al, 2012. Turkey** (52)	Retrosp cohort	2004 – 2010	Women delivering between 20 – 42 wks -2^nd^ and/or 3^rd^ trimester and confirmed during CD	PP (123) NPP (11911)	PP: Partially or completely covering the IO	PTB (n/N)
**Zaitoun et al, 2011. Egypt** (53)	Retrosp cohort	Jan 2008 – Jun 2010	-Women with PP between 28 – 36 wks -Confirmed 36 – 37 wks	PP (54)	PP: completely covering the IO	PTB (n/N) GAD (Mean, SD)
**Zlatnik et al, 2007. USA** (54)	Retrosp cohort	Jan 1980 – Dec 2001	-Women delivering >24 wks -2^nd^ trimester	PP (230) NPP (38,310)	Not low-lying placentas	PTB (n/N) GAD (Mean, SD)

CD, cesarean delivery; PP, placenta previa; LLP, low-lying placenta; NPP, no placenta previa; wks, weeks; PTB, preterm birth; GAD, gestational age at delivery; IO, internal os.

ICD 9 641.1: Low-lying placenta NOS or with hemorrhage (intrapartum)/Placenta previa: incomplete, marginal, partial, total with or without hemorrhage; 641.0: without hemorrhage.

ICD 10 O44.0: Complete placenta previa without bleeding; O44.1: Complete placenta previa with hemorrhage, unspecified trimester; O44.2: Partial placenta previa without hemorrhage; O44.3: low lying placenta with hemorrhage, unspecified trimester; O44.9 placenta previa without specification. SD, standard deviation.

### Assessment of risk of bias

The results of the quality assessment are shown in the **Supplementary Information 3**. Overall, the quality of included cohort and case-control studies was moderate to high. In general, the moderate score was due to the fact that there was no control group in the relevant cohort study. Within the included cohort studies, there may be publication or small study bias, as asymmetry was observed in the funnel plot of **Figure 4A**. In contrast, only 1 figure could be examined, given that fewer than 10 studies were included for the other comparisons.

### Synthesized findings

**Figure 2:**
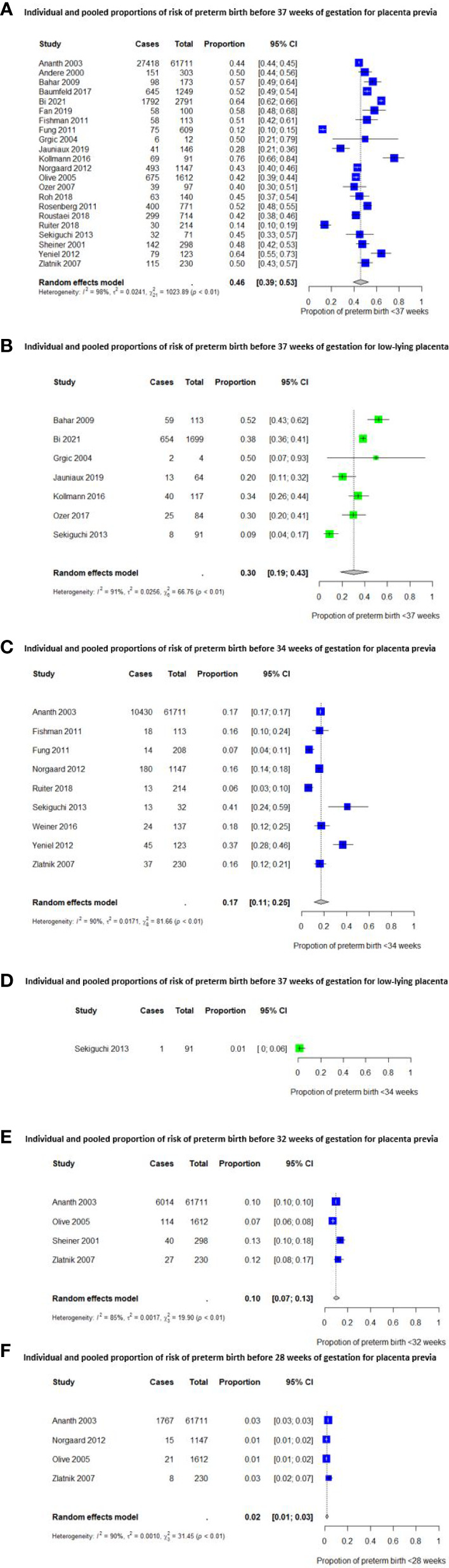
Individual and pooled proportions of risk of preterm birth before 37, 34, 32 and 28 weeks of gestation.

The pooled proportions of preterm birth for women with a placenta previa and low-lying placenta are shown in **Figure 2**. The pooled proportions before 37 weeks of gestation were 46% (95% CI [39 – 53%]) (20 studies) (4, 32, 34, 35, 37–50, 52, 54) for placenta previa and 30% (95% CI [19 – 43%]) (6 studies) (35, 40–42, 45, 49) for low-lying placenta, respectively. The proportions in these groups were 17% (95% CI [11 – 25%]) (9 studies) (32, 38, 39, 43, 48, 49, 51, 52, 54) and 1% (95% CI [0 – 6%]) (1 study) (49) respectively, for preterm birth before 34 weeks. For preterm birth before 32 and 28 weeks of gestation, studies reported only on women with a placenta previa. The pooled proportions were 10% (95% CI [7 – 13%]) (4 studies) (32, 44, 50, 54) and 2% (95% CI [1 – 3%]) (4 studies) (32, 43, 44, 54), respectively.

**Figure 3:**
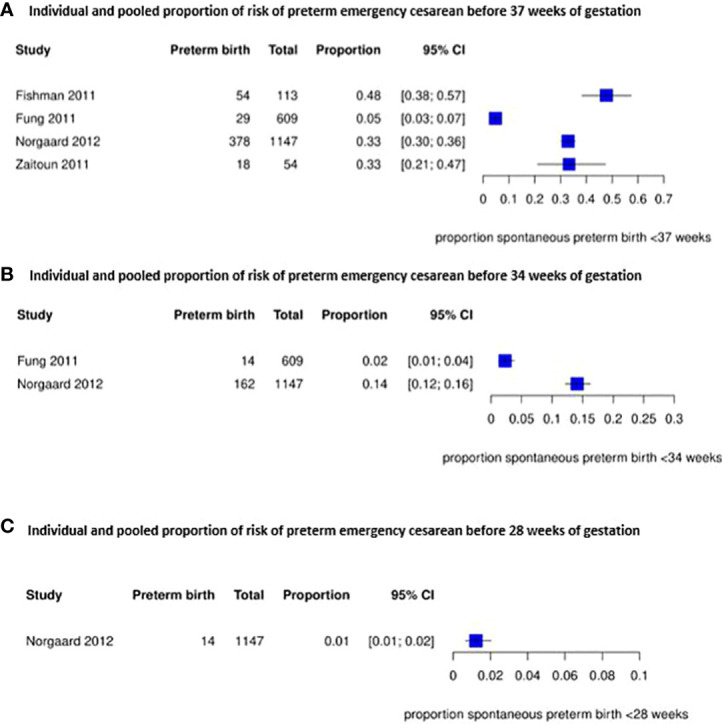
Individual and pooled proportion of risk of preterm emergency cesarean section before 37, 34 and 28 weeks of gestation for placenta previa.

The risk of a preterm emergency cesarean in women with a placenta previa is shown in **Figure 3**. For this outcome we could not pool the results due to heterogeneity between the included studies, but proportions ranged from 5% to 48% before 37 weeks (4 studies) (38, 39, 43, 53), from 2% to 14% before 34 weeks (2 studies) (39, 43) and was 1% before 28 weeks of gestation (1 study) (43). Another study reported a higher risk of preterm emergency cesarean section before 34 weeks of gestation but no higher risk of a preterm emergency cesarean before 37 weeks of gestation in women with a placenta previa (39).

**Figure 4:**
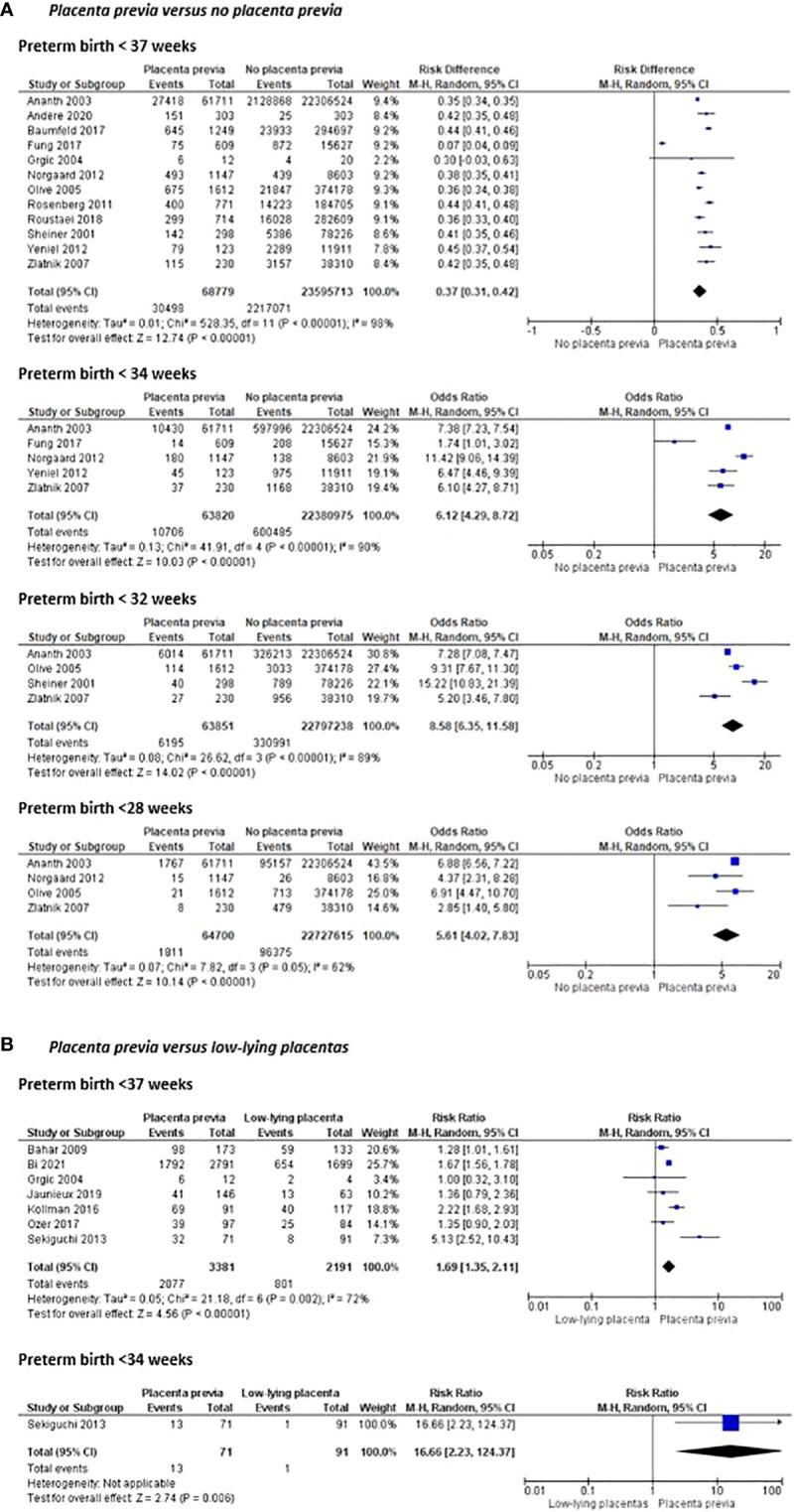
Risk of preterm birth before 37, 34, 32 and 28 weeks of pregnancy and mean gestational age at delivery between women with placenta previa and women without placenta previa or between women with placenta previa and women with a low-lying placenta.

Women with placenta previa were more likely to have a preterm birth before 37 weeks of gestation (risk difference 0.37 (95% CI [0.31-0.42]) and before 34, 32, and 28 weeks of gestation (OR 6.12 (95% CI [4.29-8.72]), OR 8.58 (95% CI [6.35 – 11.58]) and OR 5.61 (95% CI [4.02-7.83]) respectively) than women without placenta previa (**Figure 4**). Compared to women with al low-lying placenta, women with a placenta previa were also more likely to have preterm birth before 37 and 34 weeks of gestation (OR 1.69 (95% CI [1.35–2.11]) (**Figure 4**).

### Interventions to prevent preterm birth in women with a placenta previa

The interventions to prevent preterm birth were reported in nine studies (55–63). **Table 2** shows the baseline characteristics for the intervention studies.

**Table 2:** Article characteristics for studies on preventive interventions.

Author, year + country	Study design	Period	Study population	Intervention: I (N) vs control: C (N)	GA at inclusion	Outcome of interest
**Arias et al, 1988. USA (** 55 **)**	RCT	Jul 1983 – Nov 1986	Women with PP and blood loss	I: Cerclage (McDonald) (13) (+tocolytics) C: Expectant (12) (+bed rest, hospital admission, tocolytics, corticosteroids)	24-30	GAD (mean, SD) POG (mean, SD)
**Barinov et al, 2018. Russia**	RCT	2014-2016	Women with PP and a high risk of preterm delivery	I: Pessary + progesterone (81) C: Progesterone (136)	18-20	PTB (n/N)
**Chattopadhyay et al, 2015. India (** 57 **)**	RCT	Jan 2013 – Dec 2014	Women with PP/LLP + blood loss	I: Progesterone (50) C: placebo (50) Both: 2/week im until 37 wks	28-34	GAD (mean, SD) POG (mean, SD)
**Cobo et al, 1998. Colombia (** 58 **)**	RCT	Oct 1990 – Ma 1995	Women with PP	I: Cerclage (McDonald) (18) C: Expectant (18) Both: bedrest, tocolytics, corticosteroids	24-30	GAD (mean, SD) POG (mean, SD)
**Jaswal et al, 2006. India (** 59 **)**	RCT	Dec 1996 – Jun 1999	Women with PP or LLP and vaginal bleeding	I: Cerclage (McDonald) (18) C: Expectant (19) Both: bedrest, tocolytics, corticosteroids	20-34	GAD (mean, SD) POG (mean, SD)
**Sadauskas et al, 1982. Germany (** 60 **)**	RCT	–	Women with PP (a)symptomatic	I: Cerclage (62) (+tocolytics) C: Expectant (68)	16-36	POG (mean, SD) PTB (n/N)
**Shaamash et al, 2019, Egypt**	RCT	Apr 2016 = Mar 2017	Women with PP (a)symptomatic	I: Progesterone until 37 wks or delivery (54) C: Expectant (52)	24-28	GAD (mean, SD) PTB (n/N)
**Singh et al, 2015. India (** 62 **)**	RCT	–	Women with PP (a)symptomatic	I: Progesterone (40) C: placebo (40) Both: 2/week im until 37 wks or delivery	<34	GAD (mean, SD) POG (mean, SD)
**Stafford et al, 2019. USA (** 63 **)**	RCT	Nov 2016 – Jun 2018	Women with PP asymptomatic	I: Pessary (7) C: Expectant (10)	22-32	GAD (mean, SD)

POG, prolongation of gestation; GAD, gestation at delivery; wks, weeks; PP, placenta previa; LLP, low-lying placenta; 17-AHPC, 17 α-hydroxyprogesterone. SD, standard deviation.

**Figure 5:**
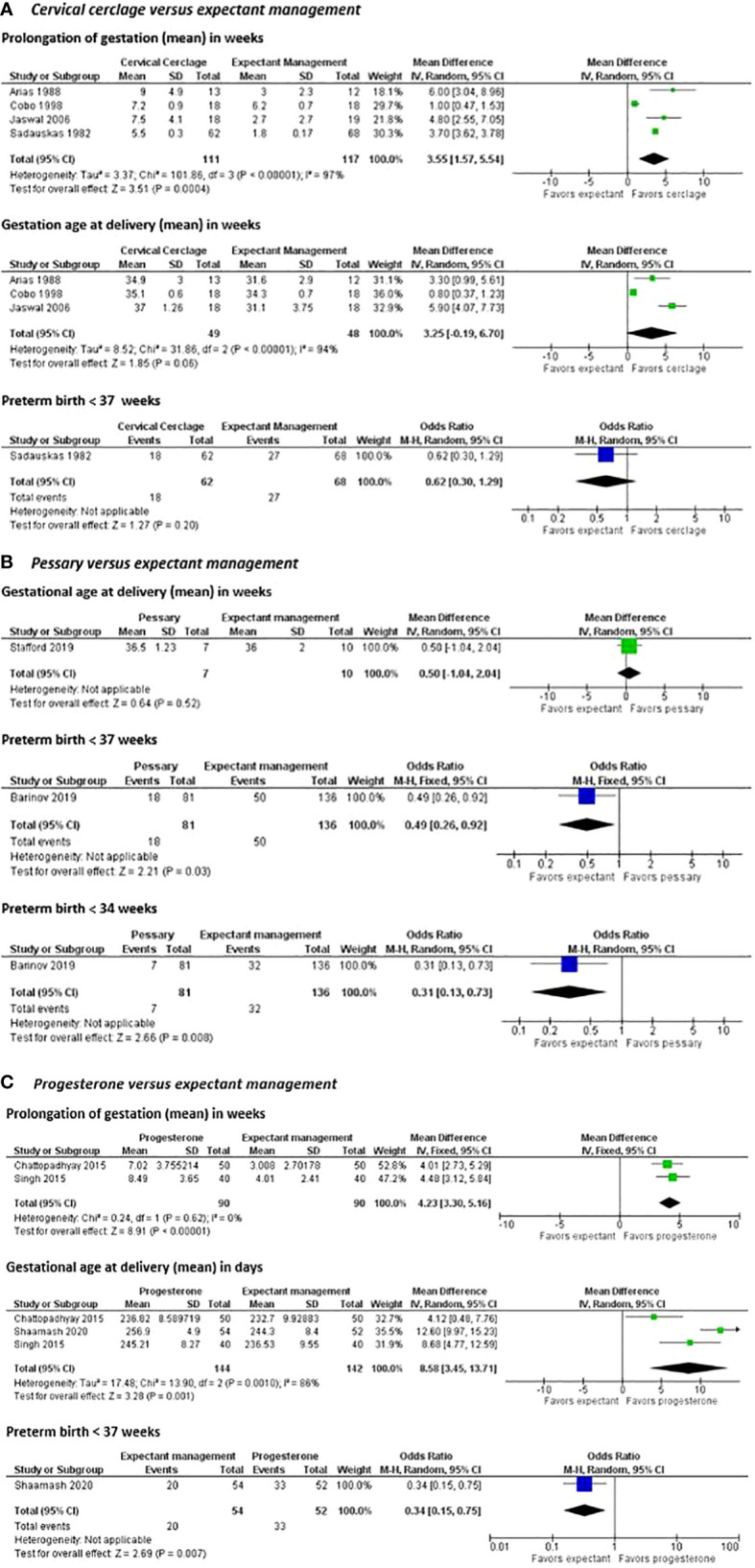
Preventive interventions for preterm birth in women with a placenta previa or low-lying placenta.

*Progesterone –*Three studies investigated the use of intramuscular progesterone in women with a placenta previa. No intervention studies were found in which vaginal progesterone was used as a treatment. All studies reported on the mean gestational age at delivery, which was significantly higher in the group of intramuscular progesterone. The pooled effect on the use of intramuscular progesterone showed a significant prolongation of gestation in favor of the women treated with progesterone in two studies (**Figure 5**) (57, 61, 62). One study showed a lower percentage of preterm birth in women using intramuscular progesterone compared to the non-progesterone group (37 vs 63% PTB P=0.007). Subsequently, in this study the mean number of bleeding attacks was significantly less in women with intramuscular progesterone (49.1 vs 67.3% P<0.001) (61).

*Pessary –* Two studies reported on a pessary as preventive intervention for women with a placenta previa. However, the first study reported the risk of preterm birth before 37 and 34 weeks, and the other study reported the mean gestational age at delivery in the two groups. Therefore, the results of the studies could not be pooled (56, 63).

Individually, the first study showed no significant difference in the mean gestational age at delivery between the group with a cervical pessary and the group with expectant management (36.5 (SD 1.23) vs. 36.0 (SD 2.00) weeks, p=0.032). However, the study was underpowered as the trial was stopped early before completion, secondary to slow enrolment and withdrawal of financial support by the sponsor. They did show however, that the number of antepartum admissions for bleeding was twofold higher in women randomized in the expectant management group (3 vs 8 admissions, not significant), suggesting that a cervical pessary placement in women with a placenta previa is associated with reduction of antepartum bleeding leading to preterm delivery (63).

The second study showed that in women with a placenta previa, already treated with progesterone because of a high risk of preterm birth due to other reasons, additional therapy of a pessary significantly reduced preterm delivery < 34 weeks (8.6 vs 23.5% P=0.031). Moreover, the use of a pessary in pregnancies with a placenta previa resulted in a three-fold reduction of the risk of bleeding during pregnancy and/or delivery (11.3 vs 33.1% bleeding, p=0.006) (56).

*Cerclage –* Four studies compared a cerclage with expectant management in women with a low-positioned placenta. The pooled effect of all four studies comparing cerclage with expectant management was in favor of cerclage considering the prolongation of gestation (mean difference of 3.55 weeks in favor of cerclage, 95% CI [1.57 – 5.54]) (55, 58–60). However, the pooled effect of the three studies reporting gestational age at delivery did not show any significant difference (mean difference 3.25 weeks in favor of cerclage, 95% CI [-0.19 – 6.70]) (55, 58, 59). Also the 1 study reporting odds of preterm birth before 37 weeks of gestation did not show any effect either (**Figure 5**) (60).

## Discussion

### Main findings

This review found a high risk of preterm birth across all gestational ages for women with a placenta previa and in women with a low-lying placenta. More specifically, a higher risk of preterm birth was found in women with placenta previa compared to women with a low-lying placenta and compared to women without placenta previa. Three interventions for the prevention of preterm birth were investigated of which cerclage, pessary and intramuscular progesterone might have benefit, but data in this population are lacking and inconsistent, so that solid conclusions about actual effectiveness cannot be drawn.

### Strengths and limitations

First, a major strength of this study is the broad search that was performed thereby including studies originated from various countries without a restriction for publication year. Second, our findings are in line with a review dating from 2015 reporting risks for preterm birth of 44% and 27% for placenta previa and low-lying placentas, respectively (8). However, we were able to include more descriptive studies of placenta previa and of low-lying placenta. Additionally, we analyzed preterm birth risk at different gestational ages providing more detailed information. Since gestational age at birth is inversely correlated with neonatal morbidity and mortality, exclusively using results of preterm birth before 37 weeks does not reflect the magnitude of the problem in this group, e.g. a birth at 28 weeks does not hold the same fetal risks as a birth at 36 weeks of gestation (26). Besides, we did not only compare women with placenta previa with women without placenta previa, but also with women with a low-lying placenta. Another strength is our strict criteria considering the definition of a placenta previa or a low-lying placenta in terms of distance to the internal os, resulting in women with better comparable defined conditions. Finally, considering the preventive interventions, we were able to give an update of the current literature, since the latest review was published in the Cochrane database in 2003, which included only 2 articles (28). We added 7 more articles for this sub-question, not only reporting on cerclage but also on a cervical pessary and progesterone.

Several limitations require to be commented as well. In general, the available studies on the interventions to prevent preterm birth are often more outdated, being 14 to 38 years old. Variation in equipment, technique and terminology could have impact on the interpretation of data and reliability of their conclusions. In addition, there was a large difference between the size of the included studies (ranging from 16 patients to 22 million patients) and the included patients differed greatly in gestational age. This results in high to very high heterogeneity (I^2^ ranging between 62 -98%) between the included studies, which reduces the strength of evidence. Furthermore, there is only 1 outcome for which more than 10 studies could be included, so only 1 funnel plot could also be made to examine publication bias.

Although we aimed only to narrate on the possible interventions to prevent preterm birth in women with placenta previa, it is still important to address the limitations considering these studies. The randomized controlled trials had a low risk of attribution and reporting bias as the outcome data was complete and no selective reporting occurred. However, some studies had a higher risk of selection bias due to lack of allocation concealment and lack of random sequence generation. Blinding was most of the time not possible but it was unclear if this had any influence on the quality of the study. In addition, it is unclear what the role of publication bias might have been on the results. However, there are so few available studies per meta-analysis per intervention that there is too little data to create a funnel plot to interpret whether publication bias is indeed present. Most important, there is a large heterogeneity in the included studies, since the patients are included over a wide range of gestational ages and indications for interventions are highly heterogeneous. This weakens conclusions about their effectiveness considerably. The analyses compare only small (underpowered) number of patients that can produce misleading results and some only involve a single study or two small studies - which is limiting the validity of a “meta-” analysis and ensures that a sensitivity analysis to the between-study heterogeneity has no added value. The same applies to the cohort studies, where there is also considerable heterogeneity between the studies. The small numbers of patients per cohort, the large variation in gestational age at inclusion, and the large variation in (obstetric) history seem to contribute mainly to this.

### Interpretation and implication

The well-recognized high risk of preterm birth for women with a placenta previa or low-lying placenta is comparable with or even higher than other known high-risk pregnancies, for example women with a history of spontaneous preterm birth (sPTB) have a risk of 15-30% on sPTB before 37 weeks of gestation in their index pregnancy (64). However, to this day the exact mechanisms are not unraveled. The two most plausible mechanisms seem associated with the cascade of placental detachment leading to (anticipated) antepartum blood loss and short cervical length. However, the reported risks of preterm birth in women with a placenta previa or low-lying placenta with and without antepartum blood loss differ between studies. The studies of Rosen et al. and Lam et al. contradict each other; where the first finds no difference in neonatal outcome between women with and without blood loss, the latter does, probably due to the gestational age at birth (65, 66). Our group previously evaluated factors that may predict an emergency delivery before the scheduled date in women with a placenta previa. We found that antepartum bleeding is an independent predictor for an emergency delivery in women with placenta previa, giving odds ratios of 7.5, 14 and 27 for one, two and three or more bleeding episodes, respectively (48). As for cervical length, a prospective cohort on the cervical length in women with placenta previa showed that women with a placenta previa and a cervical length of less than 30 mm, measured at 32 weeks of gestation or earlier if symptoms presented themselves, were three times more likely to delivery prematurely than women with a placenta previa and a cervical length over 30 mm. In addition, women with a placenta previa and a short cervix were more likely to develop antepartum blood loss (67).

Strong conclusions considering the interventions cannot be drawn, however we do suggest a benefit for progesterone, pessaries and cerclage. Progesterone acts primarily through maintaining uterine quiescence in the latter half of pregnancy, however the mechanism is unclear. Proximate to the onset of labor both term and preterm, progesterone activity withdrawals in the uterus. Therefore, progesterone supplementation in pregnancy may establish uterine relaxation, so it is reasonable to hypothesize that progesterone may be effective for women with a higher risk of preterm birth because of a placenta previa or low-lying placenta (57, 68, 69). The mechanism of a vaginal pessary is thought to be the correction of the utero-cervical angle by deviating the cervix posteriorly so the pressure from the uterus is redistributed (70, 71). Cervical cerclage is proven effective in high-risk pregnancies, mainly in women with cervical insufficiency, based on the additional support given by the suture. In case of a placenta previa, it has been suggested that the cerclage may reduce the tendency of the placenta to separate from the myometrium and thus preventing the blood loss that potentially start ends in preterm birth (26, 55). Three of the four studies demonstrated a significantly decreased rate of vaginal bleeding in the intervention group as compared to the control group (58–60).

However, given the previously described limitations, more profound and proper designed research of larger groups of patients, using the correct current definitions is necessary before incorporating preventive interventions in common practice.

## Conclusions

In conclusion, both women with a placenta previa and a low-lying placenta have an increased risk of preterm birth. This increased risk is consistent across all severities of preterm birth between 28-37 weeks of gestation. Women with placenta previa have a higher risk of preterm birth than women with a low-lying placenta. Progesterone, cervical pessary and cervical cerclage seem potentially effective preventive interventions for women with a placenta previa or low-lying placenta, but data in this population are lacking and inconsistent, so that solid conclusions about their effectiveness cannot be drawn.

## Data availability statement

The original contributions presented in the study are included in the article/**Supplementary Material**. Further inquiries can be directed to the corresponding author.

## Author contributions

CJ, EL, and EP designed the paper outline. JH and AS conducted a preliminary search led by CJ. JL designed and performed a systematic search in consultation with CJ. CJ and CD conducted the search and consulted CK in case of disagreement. Data collection and analyses were conducted by CJ and CK. All authors (CJ, CD, JH, AS, JL, BK, EL and EP) critically reviewed subsequent paper drafts and approved the submitted version. All authors agree to be accountable for all aspects of the work.

## Conflict of interest

The authors declare that the research was conducted in the absence of any commercial or financial relationships that could be construed as a potential conflict of interest.

## Publisher’s note

All claims expressed in this article are solely those of the authors and do not necessarily represent those of their affiliated organizations, or those of the publisher, the editors and the reviewers. Any product that may be evaluated in this article, or claim that may be made by its manufacturer, is not guaranteed or endorsed by the publisher.
