# Supplementary material for: Risk of preterm birth for placenta previa or low-lying placenta and possible preventive interventions: A systematic review and meta-analysis
Source: Front Endocrinol (Lausanne). 2022 Sep 2;13:921220. doi: 10.3389/fendo.2022.921220 (PMC9478860; doi:10.3389/fendo.2022.921220)
Supplement: Supplementary file 1 [file DataSheet_1.docx]

Appendix S1. Preferred Reporting Items for Systematic Reviews and Meta-analyses (PRISMA) statement

Appendix S2. Results quality assessment

Appendix S3. Results of included studies

**S1. Preferred Reporting Items for Systematic Reviews and Meta-analyses (PRISMA) statement**

Separate attached

**S2. Results quality assessment**

S2(A). Critical appraisal cohort studies risk assessment.

|  | Representativeness exposed cohort (*) | Selection non-exposed cohort (*) | Detection exposure (*) | Absence outcome at start study (*) | Comparability (**) | Outcome assessment (*) | Follow-up  (**) | Total | Conclusion  quality |
| --- | --- | --- | --- | --- | --- | --- | --- | --- | --- |
| Ananth ‘03 | **🟑** | **🟑** | **🟑** | **🟑** | **🟑🟑** | **🟑** | **🟑🟑** | **9** | **High** |
| Adere | **🟑** | **🟑** | **🟑** | **🟑** | **🟑🟑** | **🟑** | **🟑🟑** | **9** | **High** |
| Bahar | **🟑** | **-** | **🟑** | **🟑** | **🟑** | **🟑** | **🟑🟑** | **7** | **High** |
| Baumfeld | **🟑** | **🟑** | **🟑** | **-** | **-** | **🟑** | **🟑🟑** | **6** | **Moderate** |
| Bi | **🟑** | **-** | **🟑** | **🟑** | **🟑🟑** | **🟑** | **🟑** | **7** | **High** |
| Fan | **🟑** | **-** | **🟑** | **🟑** | **-** | **🟑** | **🟑🟑** | **6** | **Moderate** |
| Fishman | **🟑** | **-** | **🟑** | **🟑** | **-** | **🟑** | **🟑** | **5** | **Poor/ Moderate** |
| Fung | **🟑** | **-** | **🟑** | **🟑** | **🟑🟑** | **🟑** | **🟑🟑** | **8** | **High** |
| Jauniaux | **🟑** | **-** | **🟑** | **🟑** | **🟑🟑** | **🟑** | **🟑🟑** | **8** | **High** |
| Kollmann | **🟑** | **-** | **🟑** | **-** | **-** | **🟑** | **🟑🟑** | **5** | **Poor/ Moderate** |
| Olive | **🟑** | **🟑** | **🟑** | **🟑** | **🟑🟑** | **🟑** | **🟑🟑** | **9** | **High** |
| Ozer | **🟑** | **-** | **🟑** | **🟑** | **🟑🟑** | **🟑** | **🟑🟑** | **8** | **High** |
| Roh | **🟑** | **-** | **🟑** | **🟑** | **-** | **🟑** | **🟑** | **5** | **Poor/ Moderate** |
| Rosenberg | **🟑** | **🟑** | **🟑** | **-** | **-** | **🟑** | **🟑🟑** | **6** | **Poor/ Moderate** |
| Roustaei | **🟑** | **🟑** | **🟑** | **-** | **🟑🟑** | **🟑** | **🟑🟑** | **8** | **High** |
| Ruiter | **🟑** | **-** | **🟑** | **-** | **🟑🟑** | **🟑** | **🟑🟑** | **7** | **High** |
| Sekugichi | **🟑** | **-** | **🟑** | **🟑** | **-** | **🟑** | **🟑🟑** | **6** | **Moderate** |
| Sheiner | **🟑** | **🟑** | **🟑** | **-** | **-** | **🟑** | **🟑🟑** | **6** | **Moderate** |
| Weiner | **🟑** | **🟑** | **🟑** | **🟑** | **-** | **🟑** | **🟑🟑** | **7** | **High** |
| Yeniel | **🟑** | **🟑** | **🟑** | **-** | **🟑🟑** | **🟑** | **🟑** | **7** | **High** |
| Zaitoun | **🟑** | **-** | **🟑** | **🟑** | **-** | **🟑** | **🟑🟑** | **6** | **Moderate** |
| Zlatnik | **🟑** | **🟑** | **-** | **🟑** | **🟑🟑** | **🟑** | **🟑🟑** | **8** | **High** |

S2(B). Critical appraisal case-control studies risk assessment.

|  | Case definition (*) | Representativeness cases (*) | Selection of control (*) | Definition controls (*) | Comparability (**) | Detection exposure (**) | Non-response rate (*) | Total | Conclusion quality |
| --- | --- | --- | --- | --- | --- | --- | --- | --- | --- |
| Grgic | **🟑** | **🟑** | **-** | **🟑** | **-** | **🟑🟑** | **-** | **5** | **Poor/Moderate** |
| Norgaard | **-** | **🟑** | **🟑** | **🟑** | **🟑🟑** | **🟑🟑** | **🟑** | **8** | **High** |

S2b. Critical appraisal randomized controlled trials


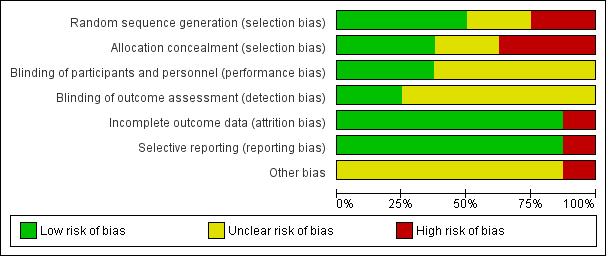


S2c. Risk of publication and small study bias assessment

Funnel plot of figure 4a. Risk difference in Preterm Birth < 37 weeks of gestation in placenta previa versus no placenta previa.


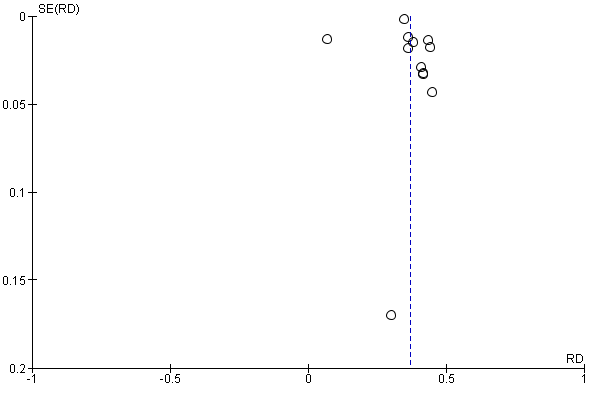


**S3. Results of included studies.**

S3(A). Risk of preterm birth.

| Author | Study groups (N) | PP (n) | NPP (n) | PP (n) | NPP (n) | PP (n) | NPP (n) | PP (n) | NNP (n) | PP mean (SD) | NPP mean (SD) |
| --- | --- | --- | --- | --- | --- | --- | --- | --- | --- | --- | --- |
|  |  | < 37 wks | | < 34 wks | | < 32 wks | | < 28 wks | | GAD | |
| Placenta previa versus no placenta previa | | | | | | | | | | | |
| Ananth ‘03 | PP (61,711)  NPP (22,306,524) | 27418 | 2128868 | 10430 | 597996 | 6014 | 326213 | 1767 | 95157 |  |  |
| Baumfeld | PP (1249)  NPP (294,697) | 645 | 23933 |  |  |  |  |  |  |  |  |
| Fung | PP (609)  NPP (15627) | 75 | 872 | 14 | 208 |  |  |  |  |  |  |
| Grgic | PP (12)  NPP (20) | 6 | 4 |  |  |  |  |  |  |  |  |
| Norgaard | PP (1147)  NPP (8603) | 493 | 439 | 180 | 138 |  |  | 15 | 26 | 36.6 (2.9) | 39.7 (2.1) |
| Olive | PP (1612)  NPP (374178) | 675 | 21847 |  |  | 114 | 3033 | 21 | 713 |  |  |
| Rosenberg | PP (771)  NPP (184,705) | 400 | 14223 |  |  |  |  |  |  |  |  |
| Roustaei | PP (714)  NPP (282,609) | 299 | 16028 |  |  |  |  |  |  | 36.4 (3.1) | 39.3 (1.9) |
| Sheiner | PP (298)  NPP (78,226) | 142 | 5386 |  |  | 40 | 789 |  |  |  |  |
| Yeniel | PP (123)  NPP (11911) | 79 | 2289 | 45 | 975 |  |  |  |  | 34 (3.9) | 37.48 (3) |
| Zlatnik | PP (230)  NPP (38,310) | 115 | 3157 | 37 | 1168 | 27 | 956 | 8 | 479 | 35.4 (2.5) | 39.1 (2.6) |
| Placenta previa versus low-lying placenta | | | | | | | | | | | |
|  | **Study groups (N)** | **PP (n)** | **LLP (n)** | **PP (n)** | **LLP (n)** | **PP (n)** | **LLP (n)** | **PP (n)** | **LLP (n)** | **PP mean (SD)** | **LLP (mean (SD)** |
|  |  | < 37 wks | | < 34 wks | | < 32 wks | | < 28 wks | | GAD | |
| Bahar | PP (173)  LLP (133) | 98 | 59 |  |  |  |  |  |  |  |  |
| Grgic | PP(12)  LLP (4) | 6 | 2 |  |  |  |  |  |  |  |  |
| Jauniaux | PP (146)  LLP (64) | 41 | 13 |  |  |  |  |  |  |  |  |
| Kollmann | PP (91)  LLP (117) | 69 | 40 |  |  |  |  |  |  |  |  |
| Ozer | PP (97)  LLP (84) | 39 | 25 |  |  |  |  |  |  |  |  |
| Sekiguchi | PP (71)  LLP (91) | 32 | 8 | 13 | 1 |  |  |  |  |  |  |
| Placenta previa proportions | | | | | | | | | | | |
|  |  | **PP (n)** |  | **PP (n)** |  |  |  |  |  | **PP mean (SD)** | **PP Median (SD)** |
|  |  | < 37 wks | | < 34 wks | |  |  |  |  | GAD | |
| Ananth ‘03 | PP (61711) | 27418 |  | 10430 |  | 6014 |  | 1767 |  |  |  |
| Adere | PP (303) | 151 |  |  |  |  |  |  |  |  |  |
| Bahar | PP (173) | 98 |  |  |  |  |  |  |  |  |  |
| Baumfeld | PP (1249) | 645 |  |  |  |  |  |  |  |  |  |
| Bi | PP (2791) | 1792 |  |  |  |  |  |  |  |  |  |
| Fan | PP (100) | 58 |  |  |  |  |  |  |  |  |  |
| Fishman | PP (113) | 58 |  | 18 |  |  |  |  |  |  |  |
| Fung | PP (609) | 75 |  | 14 |  |  |  |  |  |  |  |
| Grgic | PP (12) | 6 |  |  |  |  |  |  |  |  |  |
| Jauniaux | PP (146) | 41 |  |  |  |  |  |  |  |  |  |
| Kollman | PP (91) | 69 |  |  |  |  |  |  |  |  |  |
| Norgaard | PP (1147) | 493 |  | 170 |  |  |  | 15 |  | 36.6 (2.9) |  |
| Olive | PP (1612) | 675 |  |  |  | 114 |  | 21 |  |  |  |
| Ozer | PP (97) | 39 |  |  |  |  |  |  |  |  |  |
| Roh | PP (140) | 63 |  |  |  |  |  |  |  |  |  |
| Rosenberg | PP (771) | 400 |  |  |  |  |  |  |  |  |  |
| Roustaei | PP (714) | 299 |  |  |  |  |  |  |  |  |  |
| Ruiter | PP (214) | 30 |  | 13 |  |  |  |  |  |  |  |
| Sekiguchi | PP (71) | 32 |  |  |  |  |  |  |  |  |  |
| Sheiner | PP (298) | 142 |  |  |  | 40 |  |  |  |  |  |
| Weiner | PP (137) |  |  | 24 |  |  |  |  |  |  |  |
| Yeniel | PP (123) | 79 |  | 45 |  |  |  |  |  | 34 (3.9) |  |
| Zlatnik | PP (230) | 115 |  | 37 |  | 27 |  | 8 |  | 35.4 (2.5) |  |
| Low-lying placenta proportions | | | | | | | | | | | |
|  |  |  | **LLP (n)** |  |  |  |  |  |  |  |  |
| Bahar | LLP (133) |  | 59 |  |  |  |  |  |  |  |  |
| Bi | LPP (1699) |  | 654 |  |  |  |  |  |  |  |  |
| Grgic | LLP (4) |  | 2 |  |  |  |  |  |  |  |  |
| Jauniaux | LLP (64) |  | 13 |  |  |  |  |  |  |  |  |
| Kollmann | LLP (117) |  | 40 |  |  |  |  |  |  |  |  |
| Ozer | LLP (84) |  | 25 |  |  |  |  |  |  |  |  |
| Sekiguchi | LLP (91) |  | 8 |  |  |  |  |  |  |  |  |
| Preterm emergency cesarean section | | | | | | | | | | | |
|  | **Study groups (N)** | **PP (n)** | **NPP (n)** | **PP (n)** | **NPP (n)** | **PP (n)** | **NPP (n)** | **PP (n)** | **NPP (n)** | **PP mean (SD)** | **PP Median** |
|  |  | < 37 wks | | < 34 wks | | < 32 wks | | <28 wks | |  |  |
| Fishman | PP (113) | 54 |  |  |  |  |  |  |  |  | 34 |
| Fung | PP (609)  NPP (15627) | 29 | 661 | 14 | 208 |  |  |  |  |  |  |
| Norgaard | PP (1147) | 378 |  | 162 |  |  |  | 14 |  |  |  |
| Zaitoun | PP (54) | 18 |  |  |  |  |  |  |  |  |  |

PP placenta previa; LLP low-lying placenta; NPP no placenta previa; wks weeks; PTB preterm birth; GAD gestational age at delivery; SD standard deviation

S3(B). Preventive interventions.

| Article | Cervical cerclage | Pessary | Progesterone |
| --- | --- | --- | --- |
| Arias | -GAD: significantly higher in favor of cerclage (p=0.02)  -POG: significantly longer in favor of cerclage (p=0.006) |  |  |
| Cobo | -GAD: no significant difference  -POG: no significant difference |  |  |
| Jaswal | -GAD: significantly higher in favor of cerclage (p<0.01)  -POG: significantly longer in favor of cerclage (p<0.001) |  |  |
| Sahauskas | -POG: cerclage 38+4 days and expectant 12.8 days |  |  |
| Barinov |  | PTB: <37 weeks: no significant difference p=0.135)  <34 weeks: significantly lower in favor of the expectant group (p=0.031) |  |
| Stafford |  | GAD: No significant difference (p=0.1673) |  |
| Chattopadhyay |  |  | -GAD: significantly higher in favor of 17-AHPC (p=0.0288)  -POG: significantly longer in favor of 17-AHPC (p<0.001) |
| Shaamas |  |  | -GAD: significantly higher in favor of 17-AHPC (p< .000) |
| Singh |  |  | -GAD: significantly higher in favor of 17-AHPC (p<0.001)  -POG: significantly longer in favor of 17-AHPC (p<0.001) |
|  |  |  |  |

POG: prolongation of gestation; GAD: gestation at delivery
